# Attention Deficit Hyperactivity Disorder: A Neglected Issue in the Developing World

**DOI:** 10.1155/2014/694764

**Published:** 2014-06-25

**Authors:** J. M. Chinawa, O. I. Odetunde, Herbert A. Obu, A. T. Chinawa, Muideen O. Bakare, F. A. Ujunwa

**Affiliations:** ^1^Department of Paediatrics, University of Nigeria Teaching Hospital, PMB 01129, Enugu 400001, Nigeria; ^2^University of Nigeria Teaching Hospital (UNTH), Ituku Ozalla, PMB 01129, Enugu 400001, Nigeria; ^3^Federal Neuropsychiatric Hospital, Enugu, Nigeria

## Abstract

*Background*. Attention deficit hyperactivity disorder (ADHD) is a neglected illness in a developing country. *Objectives*. The objectives of this study are to investigate the prevalence and pattern of ADHD among children in a Nigeria. *Methods*. A structured self-administered questionnaire was used to collect information from the parents of children (and older children) who attended children outpatients' clinic during the study period. The DSM-IV-TR diagnostic criteria for attention deficit hyperactivity disorder were used. *Results*. Two hundred and seventy-three (273) out of 282 questionnaires were filled completely, giving a response rate of 96.8%. Nine (9) children fulfilled the stated criteria for ADHD giving a prevalence rate of 3.2%. There is no association between gender and ADHD (*P* = 0.784). *Conclusions*. The prevalence of ADHD in our setting is 3.2%, which is similar to that obtained elsewhere in the world.

## 1. Introduction

Attention deficit hyperactivity disorder (ADHD) is defined by features of inattention, overactivity, and impulsivity. It is the most frequently encountered childhood-onset neurodevelopmental disorder in the primary care settings [1]. Symptoms usually emerge before seven years of age. There are three major subtypes of the disorder: predominantly inattentive (ADHD-PI or ADHD-I), predominantly hyperactive-impulsive (ADHD-HI or ADHD-H), or a combination of these two subtypes (ADHD-C) [2]. ADHD has deleterious effects on school-aged children. It results in restlessness, impulsive acts, and lack of focus, which may impair school performance [3, 4].

The prevalence of ADHD is rare in the published literature in Nigeria. And ADHD prevalence seems to vary in different settings, such as in the general population versus in hospitals or in schools. Not much is known about ADHD prevalence in hospitalized Nigerian children. And there are few documented findings about the prevalence of ADHD in the outpatient setting. In other African countries, such as South Africa, Democratic Republic of Congo, or Ethiopia, the prevalence of ADHD has been reported to vary from 5.4% to 8.7% among school children. However, in the general population, ADHD has been reported in 1.5% of children. And children with possible organic brain pathology have been reported to have a prevalence of ADHD of 45.5–100% [5]. The few Nigerian studies that have been published report a prevalence of ADHD of 7.6% [6].

The prevalence of ADHD reported on other continents is variable. For instance, prevalence of ADHD in Saudi Arabian primary schools is reported to be as low as 2.7%, while that in Iran is reported to be as high as 13% [7, 8], with a predominance of the hyperactive-impulsive type. In South America, the prevalence of ADHD in children is about 6%, while in the USA it is as high as 16% [9–11]. In Germany, ADHD has been reported with a prevalence of 4.8%, while Ukraine has reported the highest incidence of ADHD to be 19% [12]. Worldwide, the prevalence of ADHD is between 5.29% and 7.1% [12].

ADHD normally affects preschool age children, although it can extend beyond childhood and adolescence into adulthood [13]. A higher prevalence is often reported in males, with the combined-type ADHD generally considered to be the most prevalent in all age groups.

Linnet et al. noted an unconfirmed link between socioeconomic factors and ADHD in children [14]. This was confirmed by Zwirs and colleagues who suggested that additional research is needed to conclusively test whether socioeconomic class is a risk factor for ADHD in at least some populations [15]. However, Döpfner and colleagues noted a higher prevalence in families of lower socioeconomic status and families from urban areas [16].

This study investigated the prevalence and pattern of ADHD in a developing country. Our hope is that this study will shed light on ADHD in a hospital setting, to help parents of affected children cope with a diagnosis of ADHD. Studies on the topic of ADHD in a developing country are available from the continent. Such studies are essential for the formulation of policies on intervention and delivery of healthcare services. The few studies that have already been conducted in Africa have mainly assessed children in a school environment. Unfortunately, many clinicians do not consider the fact that ADHD can also be diagnosed in children visiting the hospital. Many children come to the hospital, where they are diagnosed with malaria, other infections, and other common childhood illnesses. But, parents, caregivers, and even physicians sometimes miss a diagnosis of ADHD in this patient population. Thus, it may be useful to establish an area of paediatric outpatient clinics that is staffed by a child psychiatrist who can diagnose ADHD. This study will help to determine if there is a difference in the prevalence of ADHD diagnosed in a hospital versus a school setting. In addition, this study will establish baseline patterns of distribution of ADHD and will assist physicians who have a high index of suspicion to diagnose ADHD.

## 2. Methods

### 2.1. Study Area

This study was carried out in the paediatric outpatient clinic of two teaching hospitals in the Southeastern state of Nigeria: University of Nigeria Teaching Hospital (UNTH) Ituku-Ozalla, Enugu and Enugu State University Teaching Hospital (ESUTH) (both in Enugu State).

### 2.2. Study Population

Two hundred and seventy-three children and their parents who were seen on the paediatric wards of two teaching hospitals and who met inclusion criteria were consecutively recruited into our prospective study between the 1st of March and the 30th of June, 2013. The average number of patients seen was 50 per day at ESUTH and 40 per day at UNTH. However, only subjects who signed consent were included in this study.

### 2.3. Sample Size Estimation

The minimum sample size required for this study was calculated using the following formula [17]:

(1) $$\begin{array}{c} N=\frac{Z^{2}P\left(I-P\right)}{D^{2}}, \end{array}$$

where *Z* (i.e., the level of significance) = 1.96; *P* = Prevalence of children with ADHD 16% (maximal prevalence from a USA study); *D* = Tolerable error (0.05).

Using the formula above, we determined a minimum sample size of 206 patients. In anticipation of a 10% rate of attrition, the minimum sample size for our study is 270.

### 2.4. Study Procedure

A structured self-administered questionnaire was used to collect information from the parents of children, as well as older children, who presented to paediatric outpatient clinics during the study period. In a few cases in which the parents were illiterate, the questionnaire was administered by study investigators. Parents were asked to recall symptoms, from a list of criteria for the diagnosis of ADHD, exhibited by their children either at home or at school. We used the DSM-IV-TR diagnostic criteria for ADHD. According to these criteria, six of the nine symptoms in each section of inattention and hyperactivity/impulsivity must be present for at least 6 months to make a diagnosis of “combined type” ADHD [6]. Some hyperactive/impulsive or inattentive symptoms that cause impairment should be present before age of 7 years old. Other criteria we considered were that some impairment from symptoms must be present in two or more settings (e.g., at school and/or at home), and there must be clear evidence of significant impairment in social, school, or work function. Also, symptoms may not occur in the setting of a diagnosis of pervasive developmental disorder, schizophrenia, or another psychotic disorder. To avoid bias in diagnosis, the two researchers who distributed the questionnaires also explained to the parents in detail every section of the questionnaire and took a detailed clinical history of the subjects.

The paediatrician also performed a thorough physical examination of each child in the study over the course of the study. Parents of children who signed consent and who understood the questionnaire thoroughly were included in this study, while individuals with other medical or psychiatric disorders and children whose parents did not sign consent were excluded.

Each family was assigned a socioeconomic class using a recommended method, modified by Oyedeji [18]. Parents' occupations and highest level of education were assigned a score from 1 (highest) to 5 (lowest). The mean score for both parents provided a score for social class that fell within the 1–5 range. Those with a mean score <2 were further subclassified into upper class, while those with a mean score >2 were subclassified into a lower social class. For the occupation score, those in the upper social class included parents whose occupations included positions as senior public officers, large-scale traders, large-scale farmers, and professionals. Lower class occupations included artisans, primary school teachers, peasant farmers, labourers, and the unemployed. For the education score, those with a Ph.D., master's degree, bachelor's degree, or higher national diploma were categorized as upper class. Those with an ordinary national diploma, national certificate of education, technical education, grade II teachers' certificate, junior or senior secondary school certificate, primary school certificate, or no formal education were classified as lower social class [18].

It is interesting and important to investigate the prevalence of ADHD in a hospital setting because a child may actually be hyperactive with other illnesses like malaria. The physician may, thus, ignore the former disorder and focus on the later. Thus, many children come to the hospital with various degrees of hyperactivity and inattention but go unnoticed and undiagnosed.

## 3. Materials

The questionnaire used in this study is a guide for the diagnosis of ADHD, adopted from the Disruptive Behaviour Disorder Rating Scale contained in the DSM-IV [6]. This questionnaire has been validated in a Nigerian population by Ambuabunos et al. [6] in Benin City, Nigeria.

During the process of informed consent, parents or caregivers and children were informed that study participation was voluntary, that responses to the questionnaire were anonymous, and that they could withdraw from the study at any point in time if they so desired without any consequences.

This study was designed to determine the prevalence of ADHD among children presenting to Nigerian hospitals and to determine if this prevalence was different from the known school prevalence. We also aimed to determine if there was a correlation between ADHD and socioeconomic class.

### 3.1. Ethical Consideration and Consent

Ethical clearance for this study was sought from the Research and Ethical Committee of the University of Nigeria Teaching Hospital Ituku/Ozalla, Enugu. Informed consent was sought from parents/caregivers of potential subjects before enrolling them into the study.

### 3.2. Case Selection

Subjects who fulfilled the inclusion criteria were consecutively enrolled into the study.

The objectives of this study are to investigate the prevalence and pattern of ADHD in our environment.

### 3.3. Data Analysis

Data was analysed using the SPSS statistical package, version 17. The chi-square statistical test and *t*-test were used for categorical and continuous variables, respectively. The confidence interval was set at 95%.

## 4. Results

Two hundred and seventy-three out of 282 questionnaires were filled-out completely, which equated to a response rate of 96.8%. There were a total of 138 males and 144 females. This equated to a male to female ratio of 1 : 1 (*P* = 0.72). The age of study participants ranged between two- and 13-year-old, with mean age of 7.06 years old (*P* = 0.21).

The socioeconomic classes of parents are also shown in Table 1. Twenty-one (7.4%) respondents belonged to the high socioeconomic class, 145 (51.4%) respondents belonged to the middle socioeconomic class, and 116 (41.1%) respondents belonged to the low socioeconomic class.

Table 2 shows that nine children met criteria for a diagnosis of ADHD, which resulted in a prevalence rate of 3.2%. There was no association between gender and ADHD (*P* = 0.784). Among those with ADHD, eight were predominantly inattentive and one was predominantly hyperactive.

Although ADHD criteria were fulfilled more often among children >6 years old than among those <6 years old, the association between ADHD and age was not significant (*P* = 0.46).

Table 3 shows that although criteria for ADHD were fulfilled more often among children whose parents were from a low or middle socioeconomic class, ADHD did not correlate with socioeconomic class (*P* = 0.6).

## 5. Discussion

From this study, we determined that the prevalence of ADHD was 3.2%. Several reviews of the literature have reported highly variable prevalence rates for ADHD worldwide, ranging from a low of 1% to a high of almost 20% among school-age children [19, 20]. This prevalence was determined from studies conducted in a school environment. Although the causes of the variability in ADHD prevalence worldwide are unknown, geographic and demographic factors have been implicated [21]. The hospital prevalence of ADHD obtained in our study is similar to that of studies conducted in schools, where prevalence of ADHD has been reported from 1.7% to 17.8% [10]. The reason for the similarity between reported rates and ADHD prevalence in our study may be that these hospitals are commonly patronized by the majority of parents from Enugu, where the present study was performed. A debate exists as to whether ADHD might be a cultural construct [22–24]. The opinion that geographic location may influence the epidemiology of ADHD and attention deficit hyperactivity symptoms persists despite findings to the contrary in a few studies that culture and geographic location may have little to no influence on the epidemiology of ADHD worldwide [25].

It has also been argued by other experts that the variability in ADHD/HD prevalence estimates may be best explained by the use of different case definitions. They argue that the actual prevalence across geographic sites should not vary when case definitions are identical [26, 27]. Interestingly, work compiled in Africa has shown an overall prevalence of 4% [25]. This is close to our own findings.

In the present study, we also found that ADHD prevalence does not vary by gender. This is different from the results of most studies where a male preponderance has been noted [27]. Although little doubt remains that ADHD affects both genders, the literature on ADHD in females remains limited [27]. The reason why we found equivalent prevalence in both genders could be because the study was hospital-based. In addition, the sample size used may have affected the gender equality.

It is noted from this study that almost half of the children with ADHD were diagnosed before the age of six years old. In other studies, symptoms of ADHD may have been diagnosed before the age of six years old. And there were cases noted of late-onset ADHD in some studies. ADHD may affect not only children less than 6 years old, but even those up to 18 years old [27].

In our study, we had more children with ADHD-I than with ADHD-H or ADHD-C. This was confirmed by Erik in his study where he noted a high frequency of ADHD-I among children with ADHD [2]. This was also corroborated by a Nigerian study [6]. It has been reported that up to 98% of individuals diagnosed with ADHD during childhood no longer meet criteria for a diagnosis of ADHD-H at follow-up seven to eight years later [28]. In addition, in the US, children diagnosed with ADHD, when reassessed each year for eight years, were more likely to shift to a different subtype of ADHD. We did not follow our subjects over a long period of time. As such, we do not know if they changed ADHD subtype over time [29].

Although children diagnosed with ADHD were noted to come mainly from middle and low socioeconomic classes, when we correlated socioeconomic class and prevalence of ADHD, we did not find an association between socioeconomic class and ADHD. Studies in some countries have indicated that individuals from low socioeconomic class environments are 1.5–4 times more likely to meet criteria for ADHD than individuals from families from high socioeconomic classes [15]. However, other studies have not found a significant relationship between socioeconomic class and the prevalence of ADHD [28, 29].

## 6. Conclusion

We conclude that the prevalence of ADHD in our setting was similar to that in other parts of the world, and there is need for additional studies in this subregion. This is because ADHD is one of the emerging mental health problems and a neglected health issue in Africa.

## Figures and Tables

**Table 1:** Sociodemographic variables.

Characteristic	Frequency	Percent	
Sex			
Male	138	48.9	*P* = 0.72
Female	144	51.1	
Age			
<6 Years	128	45.4	*P* = 0.21
>6 Years	154	54.6	
Mother's educational level:			
Level 1	153	54.3	
Level 2	90	31.9	
Level 3	10	3.5	
Level 4	29	10.3	
Father's occupational class			
Class I	188	66.7	
Class II	24	8.5	
Class III	3	1.1	
Class IV	55	19.5	
Class V	12	4.3	
Socioeconomic class			
High socioeconomic class	21	7.4	
Middle socioeconomic class	145	51.4	
Low socioeconomic class	116	41.1	

**Table 2:** Factors influencing ADHD.

Characteristics	Has ADHD	Does not have ADHD	*X* ^2^	*P* value
(1) Sex				
Male	4 (2.9%)	134 (97.1%)	0.075	0.784
Female	5 (3.5%)	139 (96.5%)		
(2) Age				
<7 Years	3 (2.3%)	125 (97.7%)	0.545	0.460
≥7 Years	6 (3.9%)	148 (96.1%)		
(3) Socioeconomic class				
High socioeconomic class	1 (4.8%)	20 (95.2%)	1.396	0.497
Middle socioeconomic class	6 (4.1%)	139 (95.9%)		
Low socioeconomic class	2 (1.7%)	114 (98.3%)		
(4) Predominantly			
Inattentive	8	0	0
Predominantly			
Hyperactive	1	0	0
Inattention-hyperactivity combined	0	0	0

**Table 3:** Correlation between socioeconomic class and ADHD.

Correlations		Socioeconomicclass	ADHD confirmed
Socioeconomic class	Pearson correlation	1	−0.067
	Sig. (2-tailed)		0.261
	*N *	282	282

ADHD confirmed	Pearson correlation	−0.067	1
	Sig. (2-tailed)	0.261	
	*N *	282	282
